# Human Body Parts Tracking and Kinematic Features Assessment Based on RSSI and Inertial Sensor Measurements

**DOI:** 10.3390/s130911289

**Published:** 2013-08-23

**Authors:** Gaddi Blumrosen, Ami Luttwak

**Affiliations:** School of Computer Science & Engineering, Hebrew University of Jerusalem, Jerusalem 91904, Israel; E-Mail: ami.luttwak@gmail.com

**Keywords:** Body Area Network, gait analysis, daily activity, Kalman filter, RSSI

## Abstract

Acquisition of patient kinematics in different environments plays an important role in the detection of risk situations such as fall detection in elderly patients, in rehabilitation of patients with injuries, and in the design of treatment plans for patients with neurological diseases. Received Signal Strength Indicator (RSSI) measurements in a Body Area Network (BAN), capture the signal power on a radio link. The main aim of this paper is to demonstrate the potential of utilizing RSSI measurements in assessment of human kinematic features, and to give methods to determine these features. RSSI measurements can be used for tracking different body parts' displacements on scales of a few centimeters, for classifying motion and gait patterns instead of inertial sensors, and to serve as an additional reference to other sensors, in particular inertial sensors. Criteria and analytical methods for body part tracking, kinematic motion feature extraction, and a Kalman filter model for aggregation of RSSI and inertial sensor were derived. The methods were verified by a set of experiments performed in an indoor environment. In the future, the use of RSSI measurements can help in continuous assessment of various kinematic features of patients during their daily life activities and enhance medical diagnosis accuracy with lower costs.

## 1. Introduction

Acquiring a patient's kinematics and the characterization of his or her activity over time plays an important role in medicine [1]. Patient's kinematic assessment includes estimation of different body parts' position, velocity, and acceleration. The type of activity is more abstract, has a temporal characteristic, and can be divided to classes such as standing, moving from sitting to standing, walking, jumping, or lifting a bag. Human motion monitoring can help in identifying movement related problems, assist in the process of rehabilitation, design of treatment plans and follow-up monitoring [2], enable the diagnosis and treatment of numerous neurological disorders [3], and detect risk situations like falls in the elderly population [4].

Non-wearable sensing modalities are used for motion acquisition. Among these techniques, the most common ones are based on optical, electromagnetic, and ultrasonic technologies. Optical technology is usually implemented by a video recording system and is commonly used in gait analysis laboratories [5]. The estimation quality of optical methods is limited in range and azimuth, limited to high light conditions, requires calibration and *a priori* knowledge about the target people [6], heavy data streams, and computational resources for enhanced resolution. Electromagnetic based technologies can be based on narrow band, or wideband signals. A narrow band radar has been used [7] for the detection and classification of patients' movements and location based on the Doppler effect. An Ultra Wide-Band (UWB) radar, which uses a large portion of the radio spectrum, has recently been suggested for acquisition of body part displacement and motion kinematics [8]. The high EM transmission bandwidth yields accurate position location and possible material penetration. These technologies emit EM radiation to the environment and are mostly limited in range, and become not accurate in scattered environment. The Microsoft Kinect™ (Kinect), an active infra-red system [9], was recently used as a markerless method, to acquire human activity data. Reference [10] has shown accurately prediction of human pose and extract other kinematic features. Kinect still suffers from limited range and azimuth, and erroneous data. The non-wearable sensing modalities can assess human kinematics, sometimes in accurate way, but they are very much restricted to the indoor environment where the sensors are deployed, and cannot be used in outdoor environment.

Continuous monitoring of human motion while performing daily life activities has been enabled in recent years by using a Body Area Network (BAN). A BAN is a Wireless Sensor Network (WSN) with a spatially distributed autonomous sensor nodes equipped with a radio transceiver [11], located to scale of the human body dimensions [12]. Systems designed for motion estimation based on WSNs can be classified by technology, measurement metrics, and processing methods [13]. The most common motion acquisition system is an Inertial Navigation System (INS) [14]. It includes the equations that are required to derive the sensor position and orientation in a global coordination system. An INS includes an Inertial Measurement Unit (IMU), which is usually composed of miniature accelerometers, gyroscopes and sometimes a compass [15]. The IMU is usually attached to the body parts of interest and provides kinetic information about the body part movement.

In the course of accelerometer and gyroscope integration, small errors in the measurements can be accumulated and increase the tracking error over time [16]. There are several ways to compensate on the accumulation of errors over time. The repetition pattern of gait can be used to estimate the time intervals needed for carrying an efficient implementation of the strap-down integration, exclude the IMU bias, and minimize the error to less than 10% of the stride length [17] (around 5 cm per stride). Another way is to use bio-mechanics considerations. With anthropometric considerations specific to the user biomechanics—leg length and usual stride length—it is possible to limit the uncertainty growth [18]. Another way is to use the advanced filtering of the extended Kalman filter (EKF), integrated with a kinematic model [19]. The EKF model utilizes a robotic kinematic model and knowledge of human motion to estimate displacements based on gyroscope measurements. For foot displacement over an average walking distance of 3.55 m, the new EKF has an average error of 6.89 cm. Another way to reduce the error accumulation is by aggregation of other sensors [20]. All these approaches are based on exploitation of *a priori* knowledge about the type of motion, which is usually limited to repetitive motions, or limited to a specific bio-mechanics model adapted to specific sensor locations.

Received Signal Strength Indicator (RSSI) is a measurement of the signal power on a radio link [21]. It can be used for localization, link quality estimation and power control. It is part of the IEEE 802.11 protocol family and the 802.15.4 standards, and is supported by most of existing transceiver chipsets with no need for additional hardware resources. In WSN, RSSI can be calculated between set of mobile and static nodes, referred to as anchor nodes.

The available RSSI measurements can be used to estimate mobile nodes location [22]. RSSI-based tracking algorithms are usually composed of range estimation between pairs of nodes using offline calibration methods that translate the power measurements to corresponding distance between each pair of nodes [23]. Then geometric or statistical methods are applied to obtain the instant location from the range estimation [24]. Reflections of the signal from walls or from scatterers in the medium result in severe multi-paths interference at the receiving antenna. Since the calibration is usually performed offline, and is based on statistical channel realization, it cannot fully compensate for the temporal and spatial variability of the wireless medium, which reduce the position accuracy [25].

RSSI measurements have been recently used to classify movement without using a tedious calibration process. Reference [26] used a set of anchor nodes and mobile nodes attached to the arm to classify different movements by using a Support Vector Machine (SVM) technique. A Hidden Markov Model (HMM) based mechanism can be applied on the RSSI measurements for identification of different body postures [27]. These techniques are based on anchor nodes placed in known static locations, and restrict the patient location to a small area.

RSSI-based human activity systems are limited to known static locations of the anchor nodes. In addition, RSSI based systems suffer from variations in the channel, inaccurate channel modeling, tedious offline calibration process, calibration errors, packet loss, and are affected by inadequate transmit power level. As a result, today's RSSI-based tracking systems accuracy is measurable in the scale of a meter [28]. This accuracy is insufficient for bio-medical applications based on BAN where the required accuracy is in the order of a centimeter. In the work in [29] we suggested a new calibration scheme for small indoor environment, using *a priori* information about the medium. In reference [30], we showed that with high transmission rate, in close proximity, and by using the calibration scheme in [29], objects can be tracked in a scale of few centimeters. This provides an accuracy that can be used for some bio-medical applications.

In this paper, the RSSI measurements of sensor nodes that are attached to the body parts are used to track body parts, and to extract kinematic features of gait. We focus on three main techniques to exploit the RSSI measurements: track body part based on RSSI measurements only; extract kinematic features; and aggregation of RSSI measurements and other sensor modalities like IMU. For each technique, we define criteria, and tailor processing techniques. The feasibility of each technique is demonstrated and quantified for different applications in a set of experiments. A hand motion tracking was used to demonstrate tracking displacements of different body parts in scale of few centimeters. Similar methodology like in [30], was used for this purpose. We demonstrate how gait features like speed and pattern can be derived by only two sensor nodes attached to the two feet. RSSI data was aggregated with only one IMU data using a dedicated Kalman Filter. The INS was implemented by a simple strap-down integration and did not assume any assumptions on the movement and on the location of the body part. The results show that the accumulation of error was minimized using the RSSI measurements and the tracking accuracy compared with the one based only on RSSI measurements, can be improved by around 50 percent.

This paper summarizes and points out the potential of utilizing RSSI measurements. It has two main contributions. The first contribution is the technique that enables accurate tracking of body parts and extraction of motion features, by using RSSI measurements only, which have become recently available without additional cost. This can excludes the need for dedicated wearable sensor nodes. Unlike algorithms similar to the one in [26], the new suggested technique is not limited to a specific location or environment, as all the nodes can be placed on the body while in a move. The second contribution is the utilization of RSSI measurements from several nodes located at different body parts, for assessing motion features. A third contribution is a new Kalman filter model, which aggregates the RSSI data with an IMU data. The IMU data only (acceleration and angular velocity) without exploiting prior knowledge about the periodicity of the movement, or a specific kinematic model, suffers from accumulation of error over time. Aggregation of the RSSI based location estimation with the IMU data, can enable and improve IMU based tracking accuracy of different body parts without being limited to a specific body part location, to a specific type of activity, or to a specific kinematic model.

This paper is organized as follows. Section 2 describes an RSSI based tracking system for a body segment and adequate data processing techniques. Section 3, describes the system and criterion for movement classification using RSSI measurements. In Section 4, different techniques are suggested to aggregate RSSI data with other sensors. Section 5 describes the experimental set-up used. In Section 6 the experimental results are given and discussed. Section 7 summarizes the results and suggests directions for future research.

## 2. System Description

The system consists of M mobile nodes with locations of L_1_=(x_1_, y_1_, z_1_), L_2_=(x_2_, y_2_, z_1_),..,L_M_=(x_M_, y_M_, z_M_), in Cartesian coordinates and N static nodes, referred to as anchor nodes, placed at L_M+1_=(x_M+1_, y_M+1_, z_M+1_), L_M+2_=(x_M+2_, y_M+2_, z_M+2_),.., L_M+N_=(x_M+N_, y_M+N_, z_M+N_), respectively. The mobile node m'th location at instance time i is ${\text{L}}_{\text{m}}^{\text{i}}=({\text{x}}_{\text{m}}^{\text{i}},{\text{y}}_{\text{m}}^{\text{i}},{\text{z}}_{\text{m}}^{\text{i}})$. Each mobile node transmits a data packet with a known transmission power to the anchor nodes every T ms. The anchor nodes, located in the transmission range of the mobile node, calculate the received power values ${\text{P}}_{{\text{r}}_{\text{m},1}}^{\text{i}}$, ${\text{P}}_{{\text{r}}_{\text{m},2}}^{\text{i}}$,.., ${\text{P}}_{{\text{r}}_{\text{m},\text{N}}}^{\text{i}}$. Each transmitted packet is labeled with a time stamp, to synchronize between the nodes, and to recover possible packets loss.

The main goal of this paper is to derive different features from the power measurements alone, and together with other sensors' data that can be used for motion analysis. There are several sensor deployments that can be used for this purpose. One is when the anchor nodes are placed in known locations in an indoor environment, like a room, and the mobile nodes are attached to the person. From the RSSI measurements between the different anchor nodes, and the mobile node, motion features can be derived and used to track the different body part locations, and classify the person activity in indoor environment. Another setup can be when all sensor nodes are placed on different body parts. From the attenuation of radiation as measured by RSSI, other motion features can be derived. This setup is also suitable for an outdoor environment, as there are no limitations on anchor node locations. The received signal power can be modeled in a statistical manner. A common wireless channel model is the channel path-loss model [31]. The received power for mobile node m and anchor node n at time instance i in channel path-loss model is:

(1) $${\text{P}}_{{\text{r}}_{\text{m},\text{n}}}^{\text{i}}={\text{B}}_{\text{m},\text{n}}-\text{q}10{\text{log}}_{10}{\text{d}}_{\text{m},\text{n}}^{\text{i}}+\alpha_{\text{m},\text{n}}^{\text{i}},$$

Where B_m,n_ is a constant that is a function of the transmission power ${\text{P}}_{{\text{t}}_{\text{m},\text{n}}}^{\text{i}}$, the transmission wave length, and the receive and transmit antennas gains [32]; q is the channel exponent that varies between 2 (free space) and 5 (indoor with many scatterers); ${\text{d}}_{\text{m},\text{n}}^{\text{i}}$ and $\alpha_{\text{m},\text{n}}^{\text{i}}$ are the additive noise that accounts for the random effect of multi-path and for channel model inaccuracy between the i'th anchor node and the m'th mobile node.

## 3. RSSI based Tracking of a Body Segment

Tracking a body segment can be performed by attaching the mobile node to the body part of interest and using the RSSI measurements to estimate the body part's displacement over time. The anchor nodes can be located at the indoor environment, e.g., placed in the indoor environment, for example attached to the room walls, or alternatively, located on the body on relatively static body part. For example, for tracking a leg movement, the mobile node can be attached to the leg, while the anchor node can be attached to the relatively static torso. Need to note that RSSI variation is a complex phenomenon and the techniques given in this paper will be less feasible when the anchor nodes are placed in larger areas, with rich multipath, and with multiple moving objects.

Without loss of generality, we will assume that there is only one body segment which we enquire its displacements over time. For this we need only one mobile node, *i.e.*, M=1. The location of additional body parts can be tracked with additional sensor nodes using multiplexing techniques, like different frequency bands, or time slots. The maximal number of tracked body part depends on the multiplexing techniques defined by the standard, and the desired tracking accuracy.

A Minimal Mean Square Estimation (MMSE) criterion for the transformation between the RSSI measurements matrix, P_r_, and the location, is:

(2) $$\begin{array}{c} \hat{\text{f}}={\text{argmin}}_{\text{f}}\text{E}{\left[\text{L}-\text{f}\left({\text{P}}_{\text{r}}\right)\right]}^{2}\\ \text{s}.\text{t}.\left|{\text{L}}^{\text{i}+1}-{\text{L}}^{\text{i}}\right|<\delta , \end{array}$$

where L consists of Q consecutive coordinates of the mobile node; P_r_ is the K × N power measurement matrix that contains N anchor nodes power measurements over K measurements; f is a transformation of the power measurements to location; E[·] is the expected value over all stochastic sources; and δ is a bound on the difference between consecutive location estimations which are a function of transmission rate and mobile node velocity. With a high RSSI transmission rate or a low mobile node velocity, consecutive RSSI measurements imply proximate locations.

The problem is neither linear nor convex [33], thus the criterion in Equation (2) can only be solved numerically. Furthermore, an optimal transformation requires accurate statistical knowledge [34] which is not always available. Since the mobile node moves during observation time, the channel is not stationary and frequent new updates of the transformation are needed for accurate approximation.

We propose to estimate the solution to the criterion in Equation (2) by using two stages similar to [30,35]: first to approximate the range between the mobile nodes and the N anchor nodes for Q measurements interval; and then to apply geometric and statistical methods on the range approximations to obtain the instant location. Consequently, the MMSE criterion can be refined to two sub-criterions, estimation of the range between the sensor nodes, and then the estimate the location based on the range estimations:

(3) $$\hat{\text{g}}={\text{argmin}}_{\text{g}}\text{E}{\left[\text{D}-\text{g}\left({\text{P}}_{\text{r}}\right)\right]}^{2}$$

(4) $$\begin{array}{c} \hat{\text{v}}={\text{argmin}}_{\text{v}}\text{E}{\left[\text{L}-\text{v}\left(\hat{\text{D}}\right)\right]}^{2}\\ \text{s}.\text{t}.\left|{\text{L}}^{\text{i}+1}-{\text{L}}^{\text{i}}\right|<\delta , \end{array}$$

where g is the transformation that operates on the power measurements matrix P_r_ and its output is set of range estimations between the mobile node and the anchor nodes, v is the transformation that operates on the range estimations, and D̂=ĝ(p_r_) is a N × K matrix of approximated distances between the mobile nodes and the N anchor nodes over K measurements.

The RSSI-based tracking algorithm is composed of three main stages similar to [35]: calibration, range estimation, and location estimation.

The calibration scheme is performed offline, and is based on statistical models. As a result, the RSSI- based tracking accuracy is sensitive to changes in the medium, and to 2-D and 3-D changes in antenna orientations. Changes in antenna orientations, in the case of a non-isotropic antenna, where the antenna has different intensity of the radio waves in different directions, affect the tracking accuracy. Another factor that affects the tracking accuracy is the location of the sensor nodes [36]. Location estimations accuracy vary through a set of different locations in relation to the anchor nodes' positions [36]. These error factors are related to Dilution of Precision (DOP) [37]. DOP is a measure that quantifies the effect of a change sensor location and other error sources like antenna orientation on the tracking estimation accuracy.

In the second stage, the different sensor nodes' samples are synchronized, lost packets are recovered using interpolation, and the RSSI data is smoothed. Then the range between the mobile node and each anchor node is estimated by solving Equation (3) using the mapping table obtained in an offline calibration process. The calibration estimate the channel offset and exponent in a way that it reflects the channel and environment conditions.

**Figure 1.**
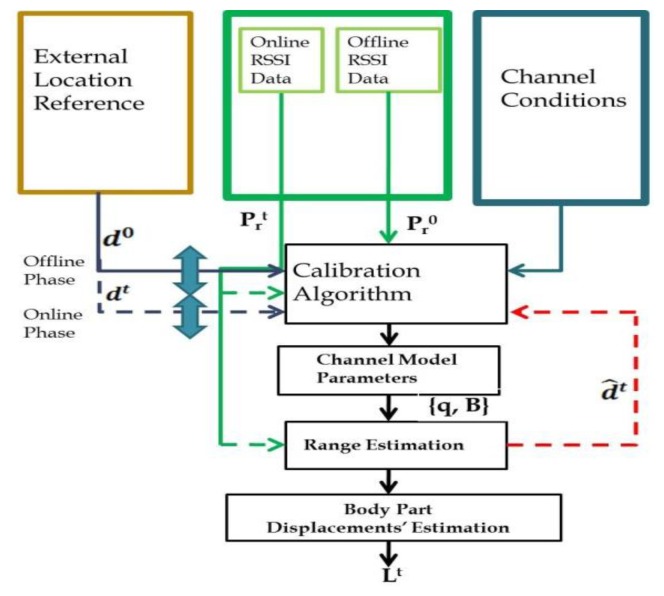
RSSI based Body part tracking scheme. Offline calibration phase produces the channel model parameters. The RSSI measurements and the channel model parameters are used to estimate the body part displacements over time.

In the third stage, the range estimations from all nodes are combined and used to solve Equation (4) The mobile node's location is estimated using the trilateration technique [38]. A median filter [39] is used to apply the continuity constraint in Equation (4). It excludes discontinuities in the location estimations and filters out location estimation errors caused by imperfect compensation for packet loss. Additional filter is applied after the median filter to mitigate over small scale multi-path fading. Figure 1 describes the different stages of the body part tracking based on RSSI measurements.

## 4. Movement Classification and Features Extraction with RSSI Data

The pattern of movement of different body parts and their kinematic features are important for medical diagnosis [40]. The RSSI measurements can be used to assess movement features. For this, the mobile nodes need to be deployed on the different body parts of interest. The anchor nodes can be deployed at different locations along the indoor environment or on other body parts.

The RSSI data can be used to classify motion, e.g., the type of gait, *i.e.*, standing, walking, or running, or to indicate the activity level. The classification can be performed directly on the RSSI measurements or on a mapping of these measurements to a feature space [41]. In some cases, a training phase, which is usually performed offline and utilizes an external reference, is needed.

Since the classifier operates directly on the RSSI measurements or on the kinematic features, the classification problem is less sensitive to sudden changes in the anchor node location, to inaccuracies in the calibration, to changes in antenna orientation and its directionality, or to packet loss, compared with the body part displacement tracking problem in the previous section. This classification capability is due to the nature of the RSSI measurements to locally preserve a pattern that is related to the movement. This pattern is relatively not affected by the typical changes in RSSI gain shifts that are caused due to channel variations and can affect the location estimation. For instance, for characterization of gait, two sensor nodes can be attached to each foot. Each node functions as a mobile node and as an anchor node (which is not in static) to the second one. Each sensor node receives transmissions of the other one, and calculates the RSSI level. Training of the system can observe some kinematic features that capture the variations of the specific setup channel, which include shadowing by body parts, creeping of the electromagnetic wave, and the antenna orientation.

Let us denoted the j'th kinematic feature by f_j_, and the operator F^i^, as the feature space mapping, f_j_ =F^j^ (P_r_). The J size feature space is defined by the group $\text{F}={\left\{{\text{f}}_{\text{i}}\right\}}_{\text{j}=1}^{\text{j}=\text{J}}$. A MMSE criterion for a classifier l is:

(5) $$\hat{\text{l}}={\text{argmin}}_{\text{l}}\text{E}{\left(\text{S}-\text{l}(\text{F})\right)}^{2}$$

where F is a K × J matrix of features, and S is a L length vector of the set of human activity classes. The mapping function estimation to classes can be more accurate by using the continuity change of the classes for consecutive times, similar to the constraint in the tracking problem in Equation (2).

Many different classifiers and feature spaces can be used. Common motion features can be the RSSI measurements raw data, the RSSI measurements distribution, in particular the mean standard deviation values, and the spectral properties of the measurements. In addition, body part kinematic features like location, acceleration, and velocity, can be used. Feature selection algorithms can be used to determine which features are more useful for each motion activity classification.

A basic feature that relate to the RSSI measurements distribution RSSI is Zero Crossing Rate (ZCR) of the RSSI measurements. The ZCR is defined as the number of zero crossings of a measurement of a reference level [42]. The mobility factor is another feature that can indicate on the patient activity level [43], can monitor patient recovery after an injury [44], and can be used to analyze freeze in PD patients [40]. It can be defined similar to [45] as the ratio of ZCR between two consecutive time instances activity:

(6) $$\text{m}{\text{f}}_{\left(\text{m},\text{n}\right)}^{\text{i}}={\text{ZCR}}_{\left(\text{m},\text{n}\right)}^{\text{i}}/{\text{ZCR}}_{\text{m},\text{n}}^{\text{i}-1},$$

where ${\text{ZCR}}_{\text{m},\text{n}}^{\text{i}}$ is the number of zero crossings in a time window of W samples of the RSSI measurements between the m'th mobile node (attached to the m'th body part) and the n'th anchor node at time instance i.

Other features can be obtained in the signal spectrum by performing a Fast Fourier Transpose (FFT) on the RSSI measurements. An important feature for analysis of periodical movements is the body part displacement's fundamental frequency. It can be obtained from the spectrum estimated by:

(7) $$\hat{\text{f}}={\text{argmax}}_{\text{f}}\left|{\text{Y}}_{\text{W}}(\text{f})\right|,$$

Where Y_W_(f) is the FFT of the RSSI measurements over a window period W, ${\left\{{\text{P}}_{{\text{r}}_{\text{m},\text{n}}}^{\text{i}}\right\}}_{\text{i}}^{\text{i}+\text{W}}$.

For the special case where the two sensors are attached to the two feet, the frequency term in Equation (7) is the inverse of the gait cycle. This technology is expected in future to be used to detect abnormalities in gait, like changes in a swing heel-off event in gait, which today is detected in gait lab by using a force plate [46].

## 5. RSSI Data Aggregation with Other Sensors

RSSI data can be aggregated together with other sensors' data using advanced statistical techniques [47]. It can be used to improve the RSSI based tracking accuracy and enable kinematic feature extraction. The criterions in Equations (2) and (5), can be extended in a way they exploit the and power measurements the other sensors' data. An extension to the MMSE criterion in Equation (2) for tracking a body part is:

(8) $$\begin{array}{c} {\hat{\text{f}}}_{\text{e}}={\text{argmin}}_{\text{f}}\text{E}{\left(\text{L}-{\text{f}}_{\text{e}}\left({\text{P}}_{\text{r}},\text{D}\right)\right)}^{2}\\ \text{s}.\text{t}.\left|{\text{L}}^{\text{i}+1}-{\text{L}}^{\text{i}}\right|<\delta , \end{array}$$

where f_e_ is the transformation that aggregate the RSSI measurements with the other sensor data measurements D.

Similarly, the criterion in Equation (5) for the extended MMSE classification method of l_e_ is:

(9) $$\begin{array}{c} {\hat{\text{l}}}_{\text{e}}={\text{argmin}}_{\text{l}}\text{E}{\left(\text{S}-{\text{l}}_{\text{e}}(\text{F},\text{D})\right)}^{2}\\ \text{s}.\text{t}.\left|{\text{S}}^{\text{i}+1}-{\text{S}}^{\text{i}}\right|<\delta_{\text{F},} \end{array}$$

where S^i^ is set of classes of interest at time instance i, and δ_F_, is a bound on the difference between consecutive classes estimations. It depends on the transmission rate and the body part velocity and acceleration.

To solve the criterion in Equation (9) advanced sensor fusion techniques like Kalman filter [48], or particle filter [49], can be used. For the common case where the additional sensor's data D is the measurements of an IMU unit, the RSSI data can be used in a simple manner to enhance the accuracy of the Kalman filter location estimations. In case of gait analysis, two sensor nodes, which include inertial sensors, are attached to each foot. In the time instance where the legs cross each other, the distance between the feet is minimal, and the RSSI measurements value is the maximal. This cross section point can be used for determination of boundaries of an SDI [17].

A more optimal approach, uses the Complementary Kalman Filter (CKF), which is also known as the Error State Kalman Filter (ESKF) [48], to estimate the instantaneous body part (mobile node) location. In the CKF, only the main error factors in the system are modeled, e.g., the sensor bias. This reduce the need to model the whole complex movement [50]. The state variables of the CKF provide additional information about body part kinematics (location, velocity, and acceleration over time). The CKF use the two independent location estimations. One is based on the RSSI based tracking system. The other on the INS system, using the IMU signals [14].

### 5.1. Sensors' Model

The sensor measurements were modeled as Gaussian process. The gyroscope signal can be modeled as:

(10) $${\text{y}}_{\text{w}}^{\text{i}}=\omega^{\text{i}}+{\text{b}}_{\text{w}}^{\text{i}}+{\text{n}}_{\text{w}}^{\text{i}},$$

Where ω^i^, ${\text{b}}_{\text{w}}^{\text{i}}$ and ${\text{n}}_{\text{w}}^{\text{i}}$ are the angular velocity vector, the related angular velocity bias, and a white Gaussian noise, at instance time i, respectively. All expressed in the three dimensions sensor coordinate system.

Similarly, the accelerator signal is modeled as:

(11) $${\text{y}}_{\text{a}}^{\text{i}}={\text{a}}_{\text{B}}^{\text{i}}-{\text{g}}_{\text{B}}+{\text{b}}_{\text{a}}^{\text{i}}+{\text{n}}_{\text{a}}^{\text{i}},$$

Where ${\text{a}}_{\text{B}}^{\text{i}}$, g_B_, ${\text{b}}_{\text{a}}^{\text{i}}$, and ${\text{n}}_{\text{a}}^{\text{i}}$, are the acceleration, gravity, acceleration bias, and noise at instance time i, respectively. All expressed in three dimensions vectors in sensor coordinate system.

The RSSI based location in global coordinates of a body part at instance time i, as derived in Section III, can be modeled as a sum of the actual position, slowly varying shadowing factor and instantaneous white Gaussian noise:

(12) $${\hat{\text{L}}}_{\text{RSSI}}^{\text{i}}={\text{L}}^{\text{i}}+{\text{b}}_{{\text{L}}_{\text{RSSI}}}^{\text{i}}+{\text{v}}_{{\text{L}}_{\text{RSSI}}}^{\text{i}},$$

Where L^i^ is the real location, ${\text{b}}_{{\text{L}}_{\text{RSSI}}}^{\text{i}}$ is the bias error, and ${\text{v}}_{{\text{L}}_{\text{RSSI}}}^{\text{i}}$ is a white mean zero Gaussian noise.

The bias ${\text{b}}_{{\text{L}}_{\text{RSSI}}}^{\text{i}}$ is related to the shadowing effect, to the DOP, and to the directionality of the antenna. The bias between successive estimations is correlated, due to the large scale fading factor, which is modeled in Equation (1) by the noise factor $\alpha_{\text{m},\text{n}}^{\text{i}}$. This correlation can be modeled by a first order Gauss-Markov process [51] with an auto correlation function of:

(13) $${\text{R}}_{\text{b}}\left(\tau \right)=\text{E}[{\text{b}}_{\text{r}}\left(\text{t}\right){\text{b}}_{\text{r}}\left(\text{t}+\tau \right)]=\sigma_{\text{b}}^{2}{\text{e}}^{-\frac{\left|\text{t}\right|}{\tau }},$$

Where $\sigma_{\text{b}}^{2}$ is the bias variance and τ is the coherence time, which is a measure of the time that multi-path components are correlated. The coherence time τ depends on the body part velocity and the medium characteristics, and is typically in range of a second for most of activities.

The RSSI based location bias, similar to accelerometer and gyroscope biases [50], can be represented in the state space [48] as:

(14) $${\text{b}}_{{\text{L}}_{\text{RSSI}}}^{\text{i}}={\text{e}}^{-\frac{{\text{T}}_{\text{s}}}{\tau }}{\text{b}}_{{\text{L}}_{\text{RSSI}}}^{\text{i}-1}+{\text{v}}_{{\text{b}}_{\text{RSSI}}}^{\text{i}},$$

Where ${\text{v}}_{{\text{b}}_{\text{RSSI}}}^{\text{i}}$ is a zero mean white Gaussian noise.

### 5.2. CKF Implementation

The INS was implemented by a simple strap-down integration and did not assume any simplifying assumptions on the type of movement, on bio-mechanical model, or on and location of the body part like in [17–19]. The difference between the location estimations of the two systems at instance time i is:

(15) $${\text{Z}}_{\in }^{\text{i}}={\hat{\text{L}}}_{\text{INS}}^{\text{i}}-{\hat{\text{L}}}_{\text{RSSI}}^{\text{i}}$$

Where ${\hat{\text{L}}}_{\text{INS}}^{\text{i}}$, and ${\hat{\text{L}}}_{\text{RSSI}}^{\text{i}}$, are the 3-D location estimations of the RSSI, and INS systems, respectively.

The difference ${\text{Z}}_{\in }^{\text{i}}$ is modeled as a function of the errors in both measurement systems, in particular, location, velocity, and orientation errors. The CKF processes the positioning difference together with the error model to estimate the location, velocity and orientation errors to minimize the error probability. These errors are then used to correct the estimations at each time instant. Consequently, the system uses a feedback design to continuously update the inertial system estimations according to the Kalman filter corrections.

A CKF uses a state space model representation to model the relationship between the model state variables and the positioning difference predicted by the model [48]. The state vector models the main error factors of the system is:

(16) $${\text{X}}_{\in }=\left[{\hat{\text{L}}}_{\varepsilon },{\hat{\text{v}}}_{\varepsilon },{\hat{\theta }}_{\varepsilon },{\hat{\text{b}}}_{\text{a},\varepsilon },{\hat{\text{b}}}_{\text{w},\varepsilon },{\hat{\text{b}}}_{{\text{L}}_{\text{RSSI}}}\right]$$

Where L̂_ε_, v̂_ε_, θ̂_ε_, b̂_a,ε_, b̂_w,ε_ are the estimation error vectors of location, velocity, orientation, accelerometer bias, angular velocity bias, respectively, and b̂_L_RSSI__ is the RSSI estimation bias.

Using a first-order approximation of the error propagation, we can model the error model as a linear transformation system, where the error is distributed as white Gaussian variable with zero mean. Consequently, the linear CKF model can be used. The CKF equations are:

(17) $$\begin{array}{c} {\text{X}}_{\in }^{\text{i}}=\text{A}\cdot {\text{X}}_{\in }^{\text{i}-1}+{\text{w}}^{\text{i}}\\ {\text{Z}}_{\in }^{\text{i}}=\text{C}\cdot {\text{X}}_{\in }^{\text{i}}+{\text{v}}^{\text{i}}, \end{array}$$

where matrix A is the state transition matrix related to the propagation of the a-priori error state vector ${\text{X}}_{\in }^{\text{i}-1}$, and C, is the measurements matrix, C= [I_3_0 0 0 0 0 0], where I_3_ is 3 × 3 identity matrix and 0 is 3 × 3 zero matrix; w^i^, and v^i^, are the estimation model and the measurements model interference vectors at instance time i, determined by the noise covariance matrices ${\text{Q}}_{\text{w}}^{\text{i}}$ and ${\text{Q}}_{\text{v}}^{\text{i}}$, of the state error model and measurements, respectively.

The state transition matrix is derived in [52], in a similar manner to [16] and [50], and by using a first-order approximation of the error propagation is:

(18) $$\text{A}=\left[\begin{array}{c} \begin{array}{cccccc} {\text{I}}_{3} & {\text{T}}_{\text{s}}{\text{I}}_{3} & 0 & 0 & 0 & 0\\ 0 & {\text{I}}_{3} & [({\text{a}}_{\text{B}}^{\text{i}}-{\text{g}}_{\text{B}})\times ]{\text{T}}_{\text{s}} & 0 & {\text{R}}_{\text{BG}}{\text{T}}_{\text{s}} & 0\\ 0 & 0 & {\text{I}}_{3} & {\text{T}}_{\text{s}} & 0 & 0\\ 0 & 0 & 0 & {\text{I}}_{3} & 0 & 0\\ 0 & 0 & 0 & 0 & {\text{I}}_{3} & 0\\ 0 & 0 & 0 & 0 & 0 & {\text{e}}^{\frac{{\text{T}}_{\text{s}}}{\tau }}{\text{I}}_{3} \end{array} \end{array}\right],$$

where the operator × is the matrix cross product operator [53], and R_BG_ is the rotation matrix from sensor to global coordinates.

It is assumed that the noise for each state variable is uncorrelated with the noise for each other state. Hence, all non-diagonal terms of the noise matrix are zero and the diagonal terms are the variances of the random variables.

**Figure 2.**
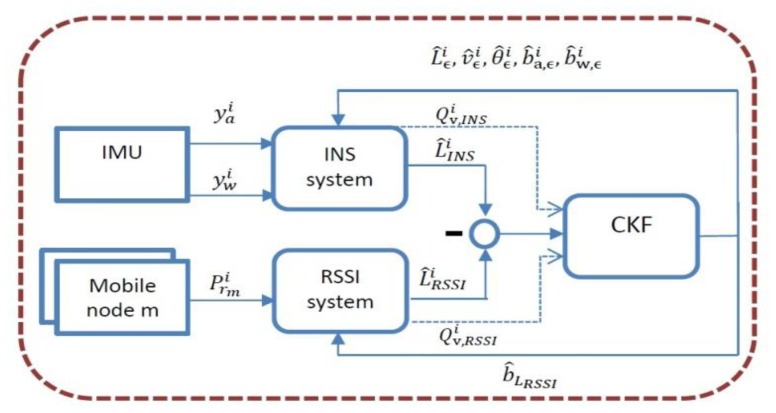
An implementation scheme of the CKF for tracking the m'th mobile node. It uses two independent estimations of location, each with different accuracies and error sources. The difference of the two estimates is fed to the CKF. The Kalman filter estimates the errors (the state vector) and corrects them continuously using a feedback loop.

An implementation scheme of the CKF is shown in Figure 2. It uses the two independent estimations of location, each with different accuracies and error sources. The IMU outputs ${\text{y}}_{\text{a}}^{\text{i}}$ and ${\text{y}}_{\text{w}}^{\text{i}}$ are fed to the INS system similar to [50]. The gravity component estimated and subtracted and the INS location estimation in global coordinates is derived. The power measurements are used to derive the RSSI based location estimation. The difference of the two estimates is fed to the CKF. The Kalman filter estimates the errors (the state vector) and corrects them continuously using a feedback loop. Need to notice, that when the RSSI data is very noisy and not reliable, and its location error estimations are high the RSSI measurements variance goes to infinity. The CKF tracking system converges then to the INS strap-down integration, and the state variables representing the biases will not be well estimated, and the system will suffer from increasing accumulation error.

The main estimation error source of ${\text{Z}}_{\in }^{i}$ is related to inaccuracy in the state variables prediction error vector, ${\text{X}}_{\in }^{\text{i}}$. The gyroscope bias and accelerometer bias lead to growing errors when the numerical integration is applied in the INS to estimate orientation, velocity or position. A small offset error in the orientation estimation accumulates over time, affects the accuracy of the gravity component extraction operation in the INS, and reduces the location estimation accuracy. The absolute location estimations obtained by the RSSI are filtered by the Kalman filter, and can help reducing the accumulation of different error factors. Other error components, which are not explicitly incorporated into the state vector, are partially modeled as part of the noise components of w^i^.

RSSI location estimations suffer from estimation bias due to an offline calibration process that does not always reflect well the dynamic changes in the channel in different locations in the environment. This process is usually performed offline and maps between the different RSSI levels and the locations using external reference such as video system. The location estimations of the different body parts is derived by solving Equation (17), and can be used as a feedback to the RSSI tracking algorithms to update in real time the calibration table in a process called Auto-Calibration (AC) [29,54]. This process can increase the accuracy of the RSSI based estimations, and the Kalman Filter estimations. For example, when moving from an environment with few scatterers to one with many, the channel exponent in channel path loss model will increase. In shadowing, the channel offset will decrease. In both cases, the RSSI based location will have a growing bias. This bias can be reduced by incorporating the Kalman location estimations in the AC process.

## 6. Experimental Section

The experimental setup was designed to show the feasibility of the proposed technologies for RSSI-based location estimation and motion classification. The experiment was composed of three main parts aimed to demonstrate different ways of exploiting the RSSI measurements. The first part demonstrated tracking of hand movement. The mobile node was attached to a moving body part (hand), while the anchor nodes were located in static known locations in the room. The second part demonstrated extraction of fundamental motion features of gait, where both mobile node and anchor nodes were placed on the two feet. The third experiment demonstrated the improvement that can be achieved in tracking a body part, by aggregation the RSSI data with inertial sensor data with relatively high bias, using CKF.

The sensor nodes were deployed in a set of distances of around a meter that are typical for BAN. The processing was partially done on the sensor nodes, and partially offline, on a remote computer. The different RSSI data packets were multiplexed in time to avoid collision. The amount and complexity of calculations is not, and in future, the calculations can be conducted on real-time on the sensor nodes.

### 6.1. Hand Movement Tracking

**Figure 3.**
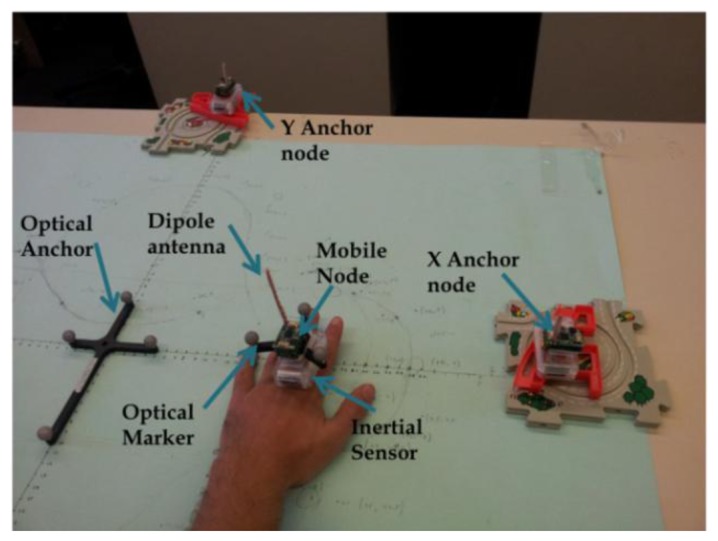
Hand movement motion tracking in 2D experiment setup. It consisted of two anchor nodes (BSN), located at *x* and *y* axes, a mobile node attached to a hand (BSN), an inertial sensor (Shimmer), used for sensor aggregation, and a reference Optical Sensor (Polaris).

The experimental setup included three sensor nodes (BSN node, Imperial College, London, UK) with an external 5 cm dipole antenna, An inertial sensor (Shimmer, Boston, MA, USA), a base station (TelosB, Andover, MA, USA), a notebook (IBM T43, Armonk, New York, NY, USA), and an optical real-time motion tracking system (Polaris, Northern Digital Inc., Ontario, Canada) used as a reference. The mobile node transmitted 802.15.4 data packets in transmission rate of 20 Hz, which is a sufficient rate to track most of daily life activities kinematic features [29]. The anchor nodes received the packets, calculated the RSSI measurements, averaged over eight symbol periods (128 μs), and sent them through the base station to the notebook for further processing. The RSSI transmitted power was around −11 dBm. The notebook was used for programming the sensors and for analyzing the results offline using Matlab Software (Matlab Inc., Natick, MA, USA;. The optical reference provided an accurate orientation and positioning information with an affective coverage of 1 square meter, a sampling rate of 60 Hz, and tracking accuracy of around 0.35 mm. The RSSI measurements and the reference Polaris system were synchronized by correlating the RSSI and the tracking system location estimations. Figure 3 describes the experimental setup used for the hand tracking in the first and third experiments. The two anchor nodes were located in the x and y axes, in coordinates of (40,0) and (0,40) centimeters, in reference to the center of the optical anchor in coordinate (0,0), respectively. In the first and third experiments the mobile node was attached to the hand and moved on the 2-D plane, which was formed by the two anchor nodes. The hand was moving in a varying speed, in an average speed of approximated 0.3 cm/s.

An offline calibration process was performed between the mobile node and each of the anchor nodes at eight different locations. Then the mobile node moved on an arbitrary path in different velocities. The maximal range between the sensor nodes was less than a meter and the channel was non-stationary due to the variations caused by the human body motion. This tried to simulate an environment that is typical for BAN.

### 6.2. Gait Features Extraction

**Figure 4.**
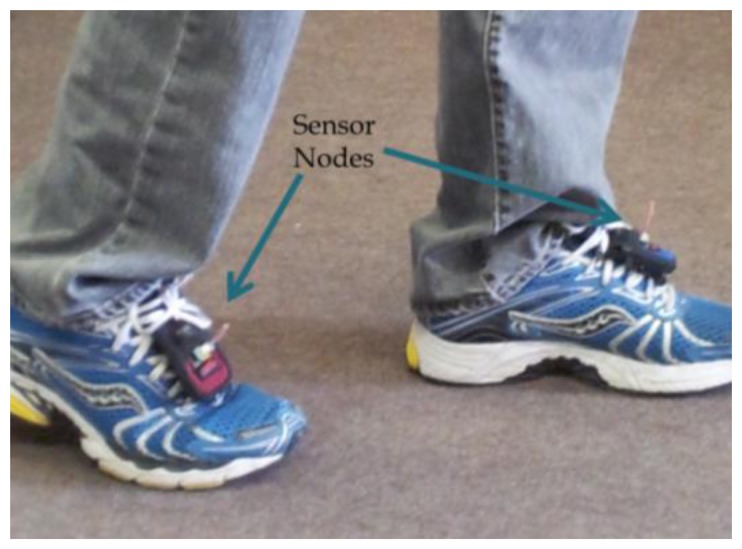
For gait classification, two sensor nodes are attached to each feet. The sensor nodes send the RSSI of each other through the base station to a processing unit to classify the gait.

In the second experiment setup, two sensor nodes (BSN nodes) were attached to each foot as shown in Figure 4. The sensor nodes included three orthogonal accelerometers producing kinematic information in 3-D. The sensor nodes calculated the RSSI of each other, and sent it for gait classification through the base station to the processing unit. Two gait scenarios were examined: walking at normal-fast pace (gait cycle of around 1 s), and running in slow pace (gait cycle of around 0.5 s). The sensor configuration was the same as the first experiment. The RSSI data was calculated in both feet in reference to the other sensor node. The features of gait cycle and ZCR, were derived from the RSSI measurements. These features were used to classify between walking or running states. Same features derived by accelerometers were used as a reference.

### 6.3. Aggregation of RSSI with Inertial Sensor

The experimental setup was similar to the hand tracking in the first experiments. The inertial sensor (Shimmer) was attached to the mobile node (BSN) in the first experiment setup as shown in Figure 3. The inertial sensor included a 3-D accelerometer and a 3-D gyroscope with sampling rate of 200 Hz. An offline calibration process was performed. For the RSSI, the calibration process was the same as in the first experiment. The calibration process for the inertial sensor included extraction of acceleration biases, and calculation of initial values of angular velocity biases. The accelerometer and gyroscope standard deviation error for each axis were 0.05 (m/s^2^), and 0.005 (Rad/s), respectively. The accelerometer and gyroscope biases was relatively high and changed over time with average value of round 0.06 (m/s^2^), and 0.03 (Rad/s) per minute in each axis, respectively. The inertial data was sent to the processing unit via Bluetooth. The RSSI data was interpolated to the inertial sensor data rate (200 Hz).The hand was moving in two patterns, circular, and zig-zag.

## 7. Results and Discussion

### 7.1. Hand Movement Tracking

**Figure 5.**
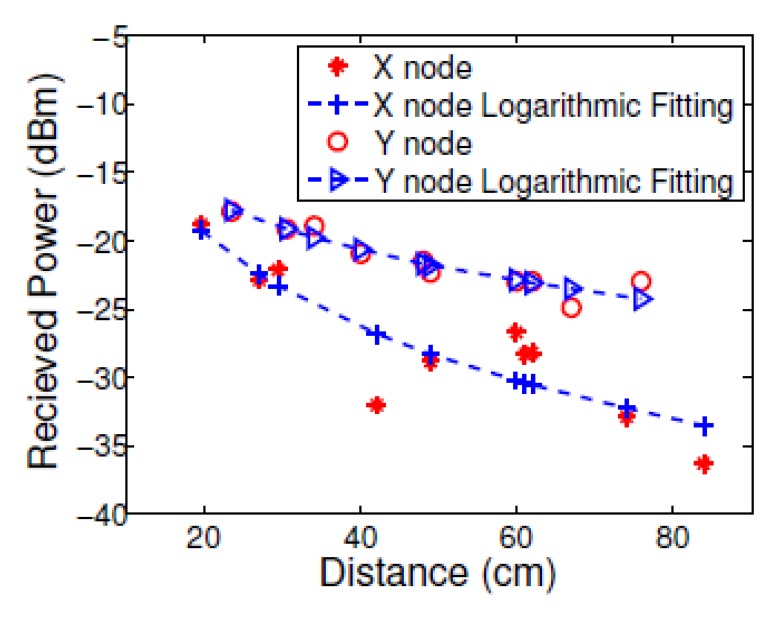
RSSI mapping between power, and distance for the x, and y nodes.

The power measurements at different locations that were taken before the tracking phase are shown in Figure 5. The channel parameters were estimated in the calibration process and used to map between the power measurements and the range between the anchor and mobile nodes similar to in [29]. The variance of the measurements compared to the logarithmic fitting, is the variance of the noise term in the path-loss model in Equation (1). The difference in gain and dynamic power range between the two nodes can be explained by the strong reflection from the body in the direction of node y that enhanced the received power and increased the power level.

We can see that the RSSI based ranging accuracy drop with distance for both nodes. This is due to the logarithmic behavior of the RSSI with distance. For distances higher than 2 m, the difference of the RSSI measurements between two proximate distances will be significantly small, compared with proximate distances. This limits the tracking resolution accuracy. Still, since the typical distance of different nodes in BAN is around a meter, the RSSI based range estimation, and correspondingly, the RSSI based tracking, will be sufficient.

**Figure 6.**
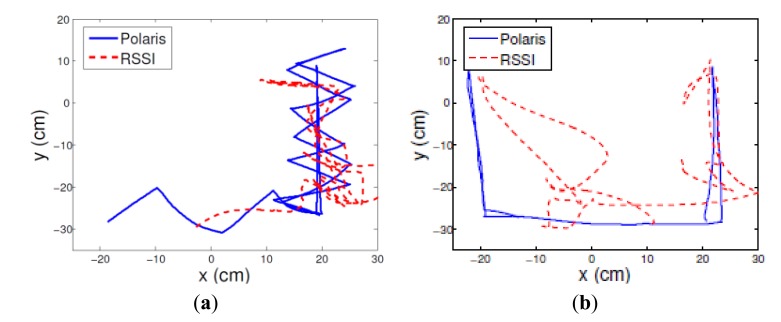
RSSI based location tracking compared to the optical reference for a hand moving in a zig-zag (**a**), and in a straight lines pattern (**b**).

The range estimations were aggregated using trilateration and an advanced filtering technique similar to the one derived in [30]. Figure 6 illustrates the tracking results of a hand moving in a zig-zag pattern, and straight horizontal and vertical lines. The location estimation mean errors were 7.88 and 10.67 cm with standard deviation mean error of 4.77 and 5.69 cm, for the zig-zag and straight patterns, respectively.

The static Cramer-Rao lower bound (CRLB) [30], calculated by the error distributions using the variance at the reference locations, were 16.37 and 15.34 cm, for the zig-zag and straight patterns, respectively. The lower standard deviation of the results is due to the exploitation of the spatial and temporal correlations and the diversity of the measurements, that were not included in the CRLB bound.

The distribution of the location estimation error is not uniform along the path. The non-uniform distribution can be explained by the dynamics of the human body motion during the hand tracking, mainly the different reflection of the radiation in different directions, which increases the variability of the medium, induces non-stationary channel conditions. In addition, the calibration, which is performed in one dimension for each sensor node, has a statistical nature, and cannot capture all of the online channel variations in 2D Future extension of the solution to 3D with non-isotropic antenna will require aggregation of online information about the antenna orientation.

In the case of tracking several body parts, multiple nodes are deployed and placed on the body. The maximal transmissions rate might decrease compared to tracking single body part, depends on the multiplexing technique used. For example, for the 802.15.4 standard, the multiplexing is performed over time. In this case, if one sensor node tracks well an object that moves in a velocity in scale of 0.5 m/s, at a transmission rate of 30 Hz, three sensor nodes, will need to cope with a transmission rate of 10 Hz. This transmission rate can still assess most of the movement in velocity in scale of 1 m/s without any significant sacrifice in performance [30].

The pattern of the movement is well captured, even when the location estimation is distorted. This indicates that the location estimations, even if is not accurate, can be useful assessment of movement pattern even in a dynamic environment, even with lower transmission rate that is needed for tracking.

### 7.2. Gait Features Extraction

**Figure 7.**
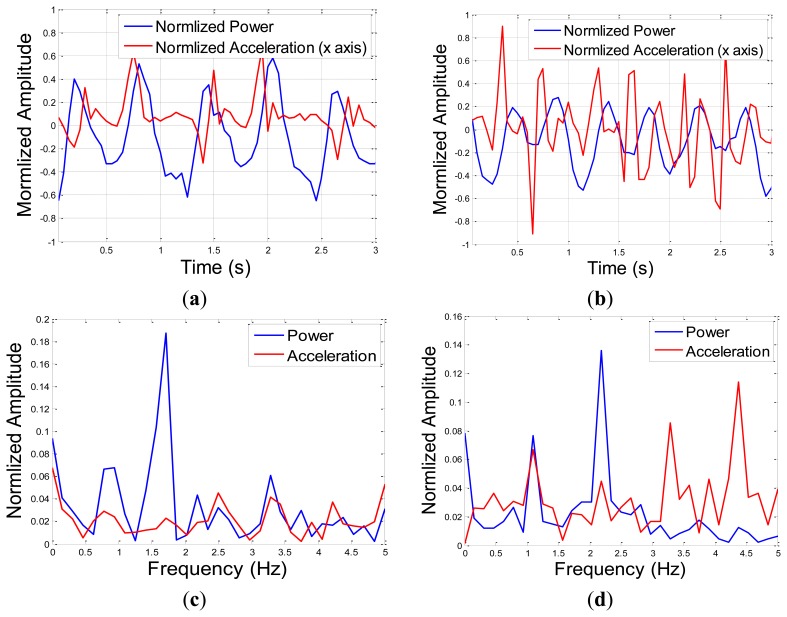
RSSI patterns of walking (**a**) and running in time (**b**) and walking (**c**) and running (**d**) in spectrum. Both RSSI and acceleration patterns have periodical pattern with a period equal to gait cycle time, but the RSSI estimations are less noisy.

The RSSI and the commonly used accelerometers' data for walking and slow-pace running in the direction of movement for three seconds is shown in Figure 7a,b, respectively. The spectrum of the RSSI and accelerometer signals for walking and slow-pace running is shown in Figure 7c,d, respectively. The values are shown in reference to the mean value and were normalized to a scale between −1 to 1, for a fair comparison.

It can be seen that in both experiments there is a periodical pattern. The repetition period is related to the gait cycle time. The gait cycle can be estimated in spectrum according to Equation (7) and is 1.6, and 2.3 Hz, for the walking and slow pace running, respectively. The zero crossing rate (ZCR) measure were calculated according to Equation (5). For the RSSI the ZCR values were 3.36 and 4.36, for the walking and the running phases, respectively, compared to 4.95 and 9.11, for the reference accelerometer measurements. The mobility factor of moving between walking and slow pace running values were 1.30 and 1.84, for the RSSI and accelerometer, respectively.

Both features seem to be statistically sufficient to distinguish between fundamental movements like standing, walking and running. The zero-crossing feature seems to preserve the pattern of the movement. The accelerometer ZCR values appear to be higher than the one of the RSSI. This is due to the nature of acceleration, as a second derivation of displacements, compared to the RSSI, which is related directly to the location estimations. The mobility factor in both cases, from stage of running to walking, was higher than 1, as expected. The lower factor in the case of RSSI can be explained by the higher low pass filtering of the RSSI measurements, and by the lower sampling rate of the RSSI samples compared to the one of the accelerometers.

The RSSI spectrum main frequency is the gait frequency, while the accelerometer frequencies include beside the gait frequency, many other frequencies. The additional frequency content of the accelerometer compared to the RSSI estimations is related to higher sensitivity of the acceleration measurements to displacements of the sensor that was moving on the foot, which was magnified as it is the second derivation of the location estimations.

The RSSI pattern might not preserve all the changes in the pattern, as in the accelerometer. Still, the RSSI location estimations, as they are related directly to range estimation, seem to capture the main movement features, and therefore might be more informative for activity level estimation.

### 7.3. Aggregation of RSSI with Inertial Sensor

The measurements noise covariance matrixes and the state initial values and the state covariance matrixes were estimated in an offline stage. The initial IMU based location and the initial CKF location estimation was set to the true location and the gyroscope bias was set to the initial value from calibration.

**Figure 8.**
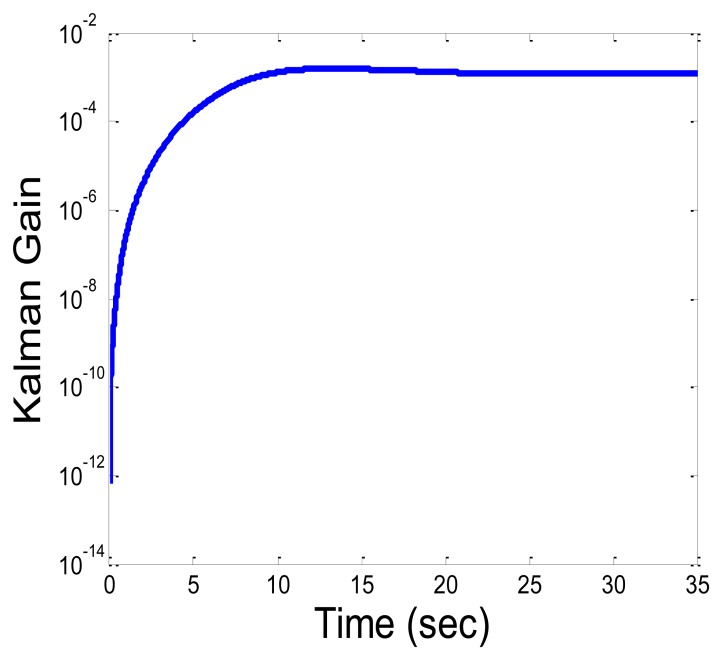
Shows the Kalman gain during experiment time.

Figure 8 describes the Kalman gain (trace of the matrix) for the circular path. From the graph, the location estimation converges to its steady state value after around 15 s. Different initial values for the covariance matrixes can change this convergence time. The location estimation based on the IMU only becomes not feasible, due to the sensor bias shift over time and the accumulation of error. The integration error in the strap-down integration in the INS grows gradually and the location estimations become not informative after few seconds having a mean and standard deviation error in scale of meters. This will be the results of the CKF, when the RSSI measurements are very noisy, and the CKF will account mainly for the output of the INS system. Need to emphasize, that there are several ways to compensate on the accumulation of error over time, and improve the INS location estimation [18–20,54]. All these approaches are based on exploitation of *a-priori* knowledge about the type of motion (periodic motions, or a specific bio-mechanics model adapted to specific sensor locations), and are not suitable for our model of one IMU unit that is located on any body part, and can move in a random path. Still, better INS performance, is expected to give better CKF results.

**Figure 9.**
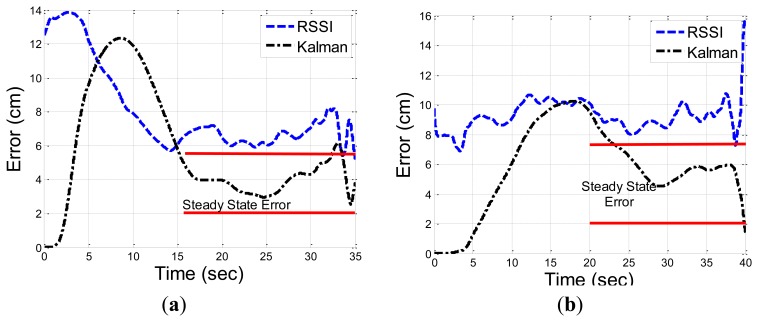
The location estimation error of the RSSI *vs.* the Kalman filter, for the circular (**a**) and zigzag patterns (**b**). The Kalman filter converges to the steady state error after approximately 15, and 20 s.

Figure 9 describes the convergence of the CKF error to its steady state error for the circular and zig-zag paths. The convergence is after 15 s, when the state variables estimations error and the corresponding Kalman gain, converge to a certain minimal value. The error in the circular pattern converge to the steady state faster, and o lower value compared to the zig-zag pattern, is lower, with shorter convergence time. This indicates that the convergence time to the steady state, depends in addition to the initial state parameter values, also on the body part speed.

**Figure 10.**
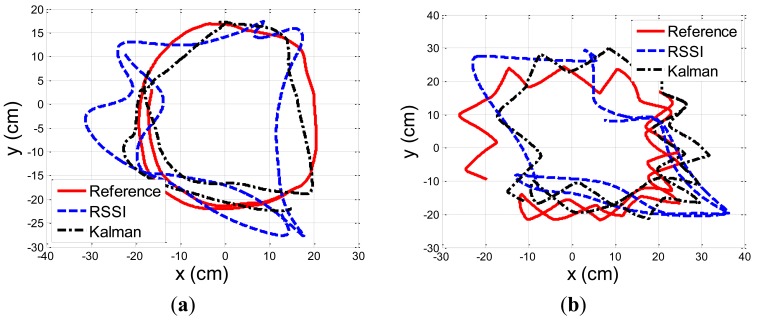
Location estimation of the RSSI, Inertial Sensor (INS), and the Kalman filter, compared to the reference (Polaris) system. Figure (**a**), and Figure (**b**) describes circular, and zig-zag motion, respectively.

Figure 10 describes the location estimation of the RSSI, and the Kalman filter, compared to the reference (Polaris) system, for a circular, and zig-zag motion patterns. The results are after convergence of the covariance matrixes to their steady state value. The RSSI based location estimations suffer from a distortion due to the offline calibration process, that provide only statistical estimations with an estimation bias that cannot compensate for the dynamic changes in the environment.

The location estimation based on the aggregation of IMU and RSSI measurements improve the RSSI based estimations significantly. The RSSI based location mean and standard error were 6.76 and 3.11 cm, and 8.11 and 5.28 cm, for the circular, and zig-zag patterns, respectively. The location estimations based on the Kalman filter in steady state, are lower by around 30%, with a mean and standard error of 4.13 and 1.96 cm, and 6.07 and 4.04 cm, for the circular, and zig-zag patterns, respectively. Table 1 summarize the results.

The location estimation based on both sensors capture the shape in a better way than based on only RSSI. In Figure 10a, the circular shape is well observed. It seems that the Kalman filter partially eliminates the effect of noisy RSSI location estimations caused by the dynamic changes in the channel that cannot be mitigated by the offline calibration. In Figure 10b, the zig-zag shape is well observed and like in the circular shape, exceptional wrong RSSI location estimations are eliminated, like the one in the right bottom corner. In the left side, wrong RSSI based location estimations are also excluded, but there is still an estimation bias of the CKF. This is due to the nature of CKF that is optimal in statistical manner, and cannot compensate for local high RSSI based location bias. The CKF, is still superior over simple low pass filtering of the RSSI location estimations, as it preserve well, despite the bias, the zig-zag pattern, that low pass filter would not resolve. In the future, an auto-calibration process, that correct in real time the RSSI location offset, can reduce the location bias, and improve the overall tracking accuracy.

These results demonstrate how the body part displacements, as estimated by the RSSI measurements, can be used to improve significantly the location accuracy of other sensors. It is achieved by continuous estimation of the measurements' biases in the CKF, and by this eliminating the growing integration error. This absolute information about location can exclude the need from additional sensor that measure global position, or orientation, like Global Positioning System (GPS), or magnetometer.

## 8. Conclusions and Future Work

RSSI measurements are unique in the sense that they are included in all BAN standards, and as such, they do not require additional hardware and software resources. Utilizing RSSI measurements of sensor nodes that are attached to the body parts of interest can be used to improve existing patient kinematics acquisition systems. Exploitation of RSSI measurements for kinematic feature assessment is a new field of research that requires extensive future research efforts. This paper's main purpose is to show feasibility of the technology, and develop the basic computational tools.

With advanced processing, and sufficient transmission rate, RSSI measurements can be used for tracking body segments within a scale of a few centimeters. This was demonstrated by tracking a hand movement. Still, many physical phenomena like shadowing, absorption by the human tissue, creeping of the electromagnetic wave along the body, affect the accuracy of the tracking information, as it is based on statistical models that cannot fully mitigate over instantaneous dynamic changes. In addition, the RSSI based tracking resolution, drop with distance, due to the nature of the wireless channel. Still, when the sensors are located in close proximity, which is the common case in BAN, the RSSI based tracking results can be sufficient to many applications.

In the case where the RSSI based tracking resolution is not adequate due to scattering, and distance limitations, the pattern of the RSSI measurements can still be utilized and be informative for motion feature assessment. We demonstrated assessment of basic kinetic feature of gait with two sensor nodes attached to the two feet. Gait pace and patterns were well assessed and verified against the traditional acceleration based assessment, in two gait speeds, normal and running.

Aggregation of the RSSI data with other common sensors, like inertial sensors, can further improve body part tracking and motion classification. The aggregation of RSSI and IMU data, can exclude the need from additional sensor that measure global position, or orientation, like GPS, or magnetometer. The tracking accuracy based only on aggregation of RSSI measurements with inertial sensor data, using an original Complementary Kalman Filter (CKF) implementation, has improved the tracking accuracy by almost 50%. This technology is expected in future to be used to detect abnormalities in gait, which today is detected in gait lab by using a force plate.

In future, the RSSI based tracking technology should be applied to track multiple body parts at different environments in 3-D. More advanced filtering, which exploits information about bio-mechanical model of the body parts, are expected to improve the system performance. Applying an online calibration process that utilizes instantaneous location estimations, can mitigate in future for part of the dynamic changes caused by changes in the environment and in sensor orientation, and improve the overall tracking estimation accuracy. More advanced classification algorithms, using more features, and feature selection algorithms, can be used to distinguish between different complex movements' classes, which are used in daily life activities.

## Figures and Tables

**Table 1.** Steady state tracking error mean and standard deviation.

**Error (cm)**	**Circular**		**Zig-Zag**	
	
	**RSSI**	**Kalman**	**RSSI**	**Kalman**
Mean	6.76	4.13	8.11	6.07
Std	3.11	1.96	5.28	4.04
